# Genomic Variation Landscape of the Model Salt Cress *Eutrema salsugineum*

**DOI:** 10.3389/fpls.2021.700161

**Published:** 2021-08-17

**Authors:** Xiaojuan Wang, Hua Rao, Jianxiang Ma, Xiaodan Chen, Guanglin Li, Guifang Zhao

**Affiliations:** ^1^Key Laboratory of Resource Biology and Biotechnology in Western China, Ministry of Education, College of Life Sciences, Northwest University, Xi'an, China; ^2^College of Life Sciences, Shaanxi Normal University, Xi'an, China; ^3^Special Economic Zone for Science and Technology Synergy, China State-Level Xixian New Area, Xi'an, China

**Keywords:** *Eutrema salsugineum*, population genomics, abiotic stress, selection, local adaptation

## Abstract

*Eutrema salsugineum* has long been used as the model for examining salt and other abiotic stress in plants. In addition to the forward genetics approaches widely used in the lab, natural variations undoubtedly will provide a rich genetic resource for studying molecular mechanisms underlying the stress tolerance and local adaptation of this species. We used 90 resequencing whole genomes of natural populations of this species across its Asian and North American distributions to detect the selection signals for genes involved in salt and other stresses at the species-range level and local distribution. We detected selection signals for genes involved in salt and other abiotic tolerance at the species level. In addition, several cold-induced and defense genes showed selection signals due to local adaptation in North America-NE Russia or northern China, respectively. These variations and findings provide valuable resources for further deciphering genetic mechanisms underlying the stress tolerance and local adaptations of this model species.

## Introduction

The development of salt-tolerant crops is becoming an urgent matter due to the increased presence of salinized soils around the world. Information on the genetic basis of salt-tolerance can be obtained by studying natural extremophiles (Amtmann et al., 2005). The salt cress, *Eutrema* (=*Thellungiella*) *salsugineum* (Brassicaceae), is a halophyte with a high tolerance to salt, cold, drought, and oxidative stress; furthermore, it is closely related to the model plant *Arabidopsis thaliana* (Bressan et al., 2001; Inan et al., 2004; Gong et al., 2005; Griffith et al., 2007; Lamdan et al., 2012; Lee et al., 2012; Zhang et al., 2013; Yu and Li, 2014). Like *A. thaliana*, salt cress is an excellent experimental system with a short life cycle, self-pollination, a small genome size and easy transformation by the floral-dip method (Yu and Li, 2014). Consequently, *E. salsugineum* has been widely used as a model to study genetic mechanisms of salt and other abiotic stress tolerance in plants (Gong et al., 2005; Griffith et al., 2007; Lamdan et al., 2012). For example, the functions of numerous ion antiporters and transporters involving salt tolerance differ greatly between *E. salsugineum* and *A. thaliana* (Zhu, 2003; Kant et al., 2006; Kumari et al., 2015). In addition, the two species differ markedly in antioxidant capacity, photosynthetic pathway and accumulation of conjugated polyamines in response to salt and other stresses (Stepien and Johnson, 2009; Pang et al., 2010). However, the part played by natural selection in causing these differences has yet to be investigated.

*E. salsugineum* is widely distributed on saline soils from central Asia to northern China and North America. Long-distance migration and formation of a geographically disjunct distribution might have occurred very recently (Wang et al., 2015, 2018) and, in turn, promoted local adaptation as observed, for instance, in *Arabidopsis thaliana* (Fournier-Level et al., 2011). In support, two commonly used ecotypes of *E. salsugineum* collected respectively from Shandong in northern China and Yukon in Canada, North America, show contrasted expressions of abiotic stress-related genes (Wong et al., 2005), which may be important in local adaptation selected by their different habitats. Annual average spring and winter temperatures are distinctly lower in Yukon (−19°C) than in northern China (4°C) (Wong et al., 2005; Griffith et al., 2007) and therefore might select for low temperature tolerance. Preliminary transcriptome analyses revealed over 39,000 SNPs differences between the salt cress populations from these two regions (Champigny et al., 2013). However, to correctly interpret these data it is necessary to characterize the evolutionary relationships between populations since many expression differences could simply reflect neutral divergence among populations (Kryvokhyzha et al., 2016).

In this study, we present analyses of the whole genomes of salt cress individuals from North America, China and Russia (Altai and Yakutsk) in order to examine genome-scale nucleotide variation across the range of the species and local adaptation. We used the obtained all high-quality genome-wide SNPs to identify genes with selection signals at the species level and local adaptation. This was facilitated by the availability of a recently developed reference genome (Wu et al., 2012; Yang et al., 2013). Re-sequencing genomes of different populations has proved highly effective for uncovering genomic signatures of selection and inferring demographic histories in model and non-model animal and plant species in model and non-model animal and plant species (Andolfatto, 2005; Olson et al., 2010; Branca et al., 2011; Huang et al., 2012; Evans et al., 2014; Li et al., 2014; Lamichhaney et al., 2015; Qiu et al., 2015; Ru et al., 2016; Sun et al., 2020; Zhao et al., 2021). Here, we first identified genes with selection signals across the species range before analyzing signals of local adaptation especially in northern China and North America-NE Russia.

## Materials and Methods

### Sample Selection and Resequencing Data Collection

Genomic nucleotide data from 90 individuals of 21 populations across the species range from central Asia to North America were obtained from the previous study (Wang et al., 2018) (Supplementary Tables 1, 2). The reference genome of salt cress, *Eutrema salsugineum*, was reported (Yang et al., 2013).

### Genome Mapping and SNP Calling

Clean reads from each sample above were aligned to the *Eutrema salsugineum* nuclear genome sequence v1.0 (Salt cress) (Yang et al., 2013). Genome mapping was conducted using BWA software with “mem” option and default parameters (Li and Durbin, 2009). The Picard package (http://picard.sourceforge.net/) was subsequently used to check for PCR duplicates. The Genome Analysis Toolkit (GATK) (Mckenna et al., 2010) was used to perform local realignment of reads to enhance the alignments in the vicinity of putative indels.

After genome mapping, The SNP calling was done for all individuals using SAMtools v1.1 (mpileup and BCFtools). Only paired aligned reads were used for SNP calling. The genotype likelihoods for each individual per site were calculated, and allele frequencies were estimated. The “mpileup” command was used to identify SNPs with the parameters “-q 30 -C 50 -S -D -Q 30 -m 2 -F 0.002 -guf.” Low-coverage depth SNPs (summing all samples) were then filtered with the vcfutils.pl in BCFtools v1.1 (Li et al., 2009) with parameters “-d 135 -D 1800” and high-quality SNPs (RMS of mapping quality ≥10, the distance of adjacent SNPs in the vicinity of indel polymorphisms ≥5 bp, Hardy-Weinberg equilibrium (HWE) P <5e-3, SNP quality ≥ 30, 3.0 ≤ quality by depth (each individual) ≤ 30, SNPs with observed heterozygosity (Ho < 0.6) were further filtered by Perl scripts. We used the obtained about 1.76 million high-quality SNPs for subsequent analysis.

### Population Phylogenetic Analyses

The software RAxML was then used to construct phylogenetic trees with the GTR-G model and 1,000 rapid bootstrapping replicates based on the Maximum Likelihood (ML) method (Stamatakis, 2015). The final Maximum Likelihood trees were viewed using FigTree (v1.4.0) (http://tree.bio.ed.ac.uk/software/figtree/).

### Screening for Selective Sweeps Across the Species Range

To identify genomic regions that might have been subject to selection during stress tolerance, we applied genetic diversity tests to the entire data from all 90 individuals. Nucleotide diversity was calculated using the standard estimate of the scaled mutation rate: the average pairwise nucleotide diversity θ_π_ (Tajima, 1989). Tajima's D was also calculated by dividing the difference between the average pairwise nucleotide diversity and the proportion of segregating sites by the square root of its SE (Tajima, 1989). We scanned the genome for regions with the highest differences using a window size of 20 kb and a step size of 10 kb. Windows that shared the lowest 5% of θ_π_ and lowest 5% Tajima's D estimates of the entire data were identified as putatively selected regions in the salt cress. Genes located in these regions were considered putatively selected genes. The above analyses were conducted using Vcftools (Danecek et al., 2011) or PERL scripts.

### Screening for Selective Sweeps for Local Adaptation

To identify genomic regions that might have been associated with local adaptation, we also applied genetic diversity tests to subpopulation data: Y1 (from northern China) and Y4 (from North America-Russia). The fixation index (*F*_ST_) (Weir and Cockerham, 1984), and nucleotide diversity (θ_π_ log-ratio Y1/Y4 and Y4/Y1) were also chosen as indicators of population differentiation. We scanned the genome for regions with the highest differences using a window size of 20 kb and a step size of 10 kb. Windows that shared the highest 5% of *F*_ST_ and highest 5% log-ratio estimates were recognized as positively selected regions in a given population. Genes located in these regions were considered putatively selected genes in local area.

### Gene Annotation Analysis

We annotated functional categories of *E. salsugineum* genes based on the corresponding *Arabidopsis thaliana* orthologs. Functional enrichment analysis of Gene Ontology (GO) was performed using the KOBAS 2.0 web server (Wu et al., 2006). The chi-squared test was used to calculate the statistical significance of enrichment and only terms with a *p*-value <0.05 were considered significant.

## Results

### Whole Genome Resequencing

We obtained re-sequenced data of the genomes of 90 *E. salsugineum* individuals spanning their worldwide geographic distributions from Wang et al. (2018). All of these data were mapped to an available reference genome (Yang et al., 2013). All high-quality single-nucleotide polymorphisms (SNPs) were further used for subsequent analysis.

**Figure 1:**
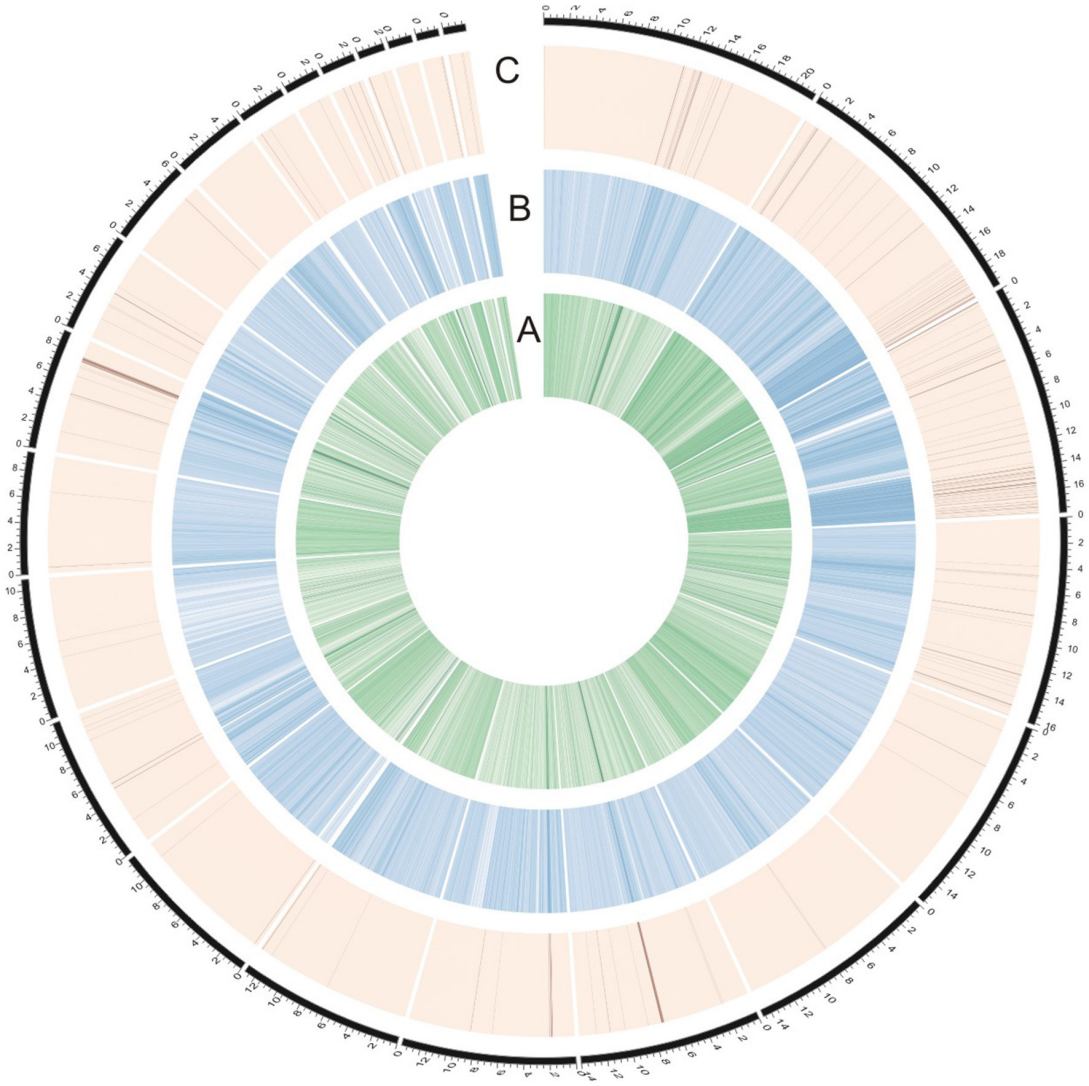
Identification of genomic regions with strong selective sweep signals in salt cress (the entire data). **(A)** The innermost circle represents θπ (green). **(B)** The middle circle represents Tajima's D (blue). **(C)** The dark lines in the outer circle represents those regions defined as selective sweeps (θπ ≤ 0.00053 and Tajima's D ≤ −1.0197). The outermost dashed black lines indicates 24 major scaffolds.

**Figure 2:**
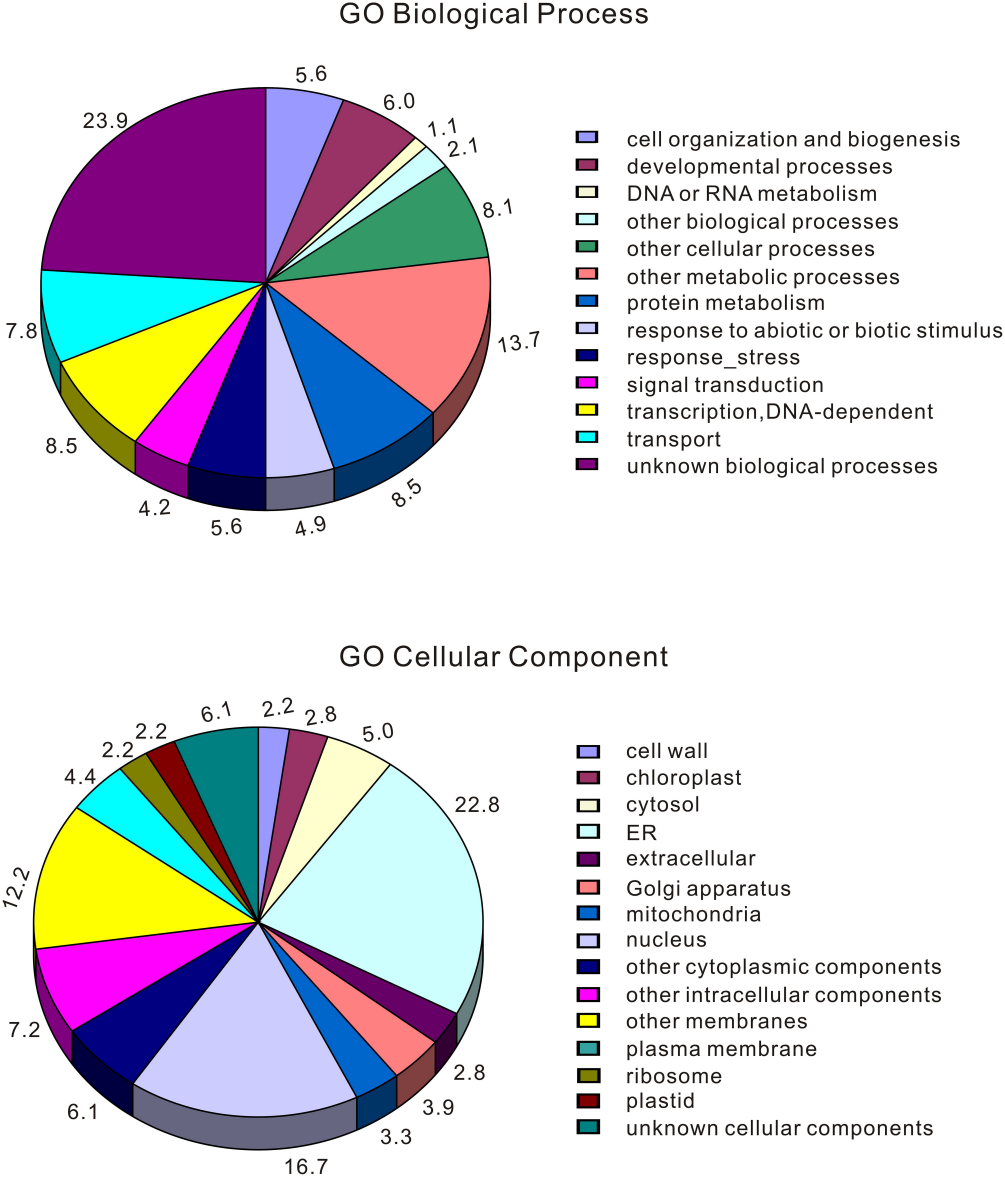
Categorization of those genes located at selected windows by Gene Ontology (the entire data). *Eutrema salsugineum* genes were assigned an *A. thaliana* locus and then categorized using TAIR automatic system. Note that a gene may be assigned to more than one biological process in the GO classification system.

To identify genomic regions that might have been subject to selection, we first combined the all populations into a single gene pool. Out of 24,039 windows of 20 kb in length sliding in 10 kb steps across the salt cress genome, 23,366 windows contain >10 SNPs and cover 97.9% of the genome (Figure 1; Supplementary Figure 1). These 23,366 windows were used to detect signatures of selective sweeps at the species level. To identify regions with selective sweep signals, we used an empirical procedure (Branca et al., 2011) and selected windows with both significantly low diversity (θ_π_) (5% right tail, where θ_π_ is 0.00053) and an excess of low-frequency variants (low D_T_) (5% right tail, where D_T_ is = −1.0197) when compared to the empirical distribution of these two statistics. This led to the identification of a total of 4.76 Mb genomic regions (1.97% of the genome, containing 262 genes) with strong selective sweep signals (Figure 1). Among these putatively selected regions, 227 genes were annotated and classified according to terms developed by the Gene Ontology Consortium (Berardini et al., 2004) and found in the TAIR database (Yon et al., 2003). Among them, 48% of genes were assigned to broad or unspecified GO categories: biological processes unknown, other cellular, other metabolic and other biological processes. We successfully classified the remaining genes to roles in transcription and signal transduction, protein, DNA or RNA metabolism, transport, development, response to abiotic or biotic stimulus and response to stress (Figure 2; Table 1; Supplementary Table 3). Of the categories with defined functions, the larger groups of biological processes were involved in response to abiotic or biotic stresses (10.5%) including stress (5.6%) and abiotic or biotic stimulus (4.9%), protein metabolism (8.5%), and transcription (8.5%) (Figure 2; Supplementary Table 3). Cell compartments clustered by GO terms showed that most of the predicted gene products were localized in the nucleus and endoplasmic reticulum (ER) (Figure 2). We identified 16 genes that were common to abiotic and biotic stresses with selection signals related to ion homeostasis, osmotic adjustment and growth regulation (Table 1). For example, seven of these (*GLP9, NDPK1, ACO2, PTR3, PYK10, NUDT7*, and *MDAR2*) are involved in response to osmotic adjustment and salt stress while some others (including *ERF13, WRKY38, DAR4*, and several disease resistance protein) are closely related to cell death and defense in plant growth regulations (Table 1).

**Table 1:** Gene ontologies (GOs) in the 16 putatively abiotic-biotic stress related genes of *E. salsugineum* (*p*-value <0.05).

	***E. salsugineum* locus**	***A. thaliana* locus**	**Brief_description**	**GO_ID**	**GO_Term**	***p*-value**
I	Thhalv10013014m	AT5G08020	RPA70-kDa subunit B (RPA70B)	GO: 0009651	Response to abiotic stimulus	0.00014
	Thhalv10013289m	AT5G07990	Transparent testa 7 (TT7)			
	Thhalv10010179m	AT3G54220	GRAS family transcription factor (SCR)			
II	Thhalv10026186m	AT4G14630	Germin-like protein 9 (GLP9)	GO: 0006970	Response to osmotic stress	0.0002
	Thhalv10029044m	AT4G09320	Nucleoside diphosphate kinase type 1 (NDPK1)	GO: 0009628	Response to salt stress	0.00014
	Thhalv10023595m	AT1G62380	1-aminocyclopropane-1-carboxylate oxidase 2 (ACO2) peptide			
	Thhalv10000830m	AT5G46050	Transporter 3 (PTR3)			
	Thhalv10001865m	AT3G09260	Glycosyl hydrolase superfamily protein (PYK10)			
	Thhalv10003061m	AT4G12720	MutT/nudix family protein (NUDT7)			
	Thhalv10013480m	AT5G03630	Pyridine nucleotide-disulfide oxidoreductase family protein (MDAR2)			
III	Thhalv10021496m	AT3G23180	HR-like lesion-inducing protein-like protein	GO: 0006952	Defense response	0.00038
	Thhalv10027189m	AT2G44840	Ethylene-responsive element binding factor 13 (ERF13)			
	Thhalv10015295m	AT5G22570	WRKY DNA-binding protein 38 (WRKY38)			
	Thhalv10000747m	AT5G45230	Disease resistance protein (TIR-NBS-LRR class) family			
IV	Thhalv10003061m	AT4G12720	MutT/nudix family protein (NUDT7)	GO: 0012501	Programmed cell death	0.0037
	Thhalv10012485m	AT5G17890	DA1-related protein 4 (DAR4)	GO: 0008219	Cell death	0.0054
	Thhalv10000747m	AT5G45230	Disease resistance protein (TIR-NBS-LRR class) family	GO: 0016265	Death	0.0054
	Thhalv10000769m	AT5G47260	Putative disease resistance protein	GO: 0006915	Apoptosis	0.025
				GO: 0006952	Defense response	0.00038

*I–IV show different groups of genes with same GO terms*.

### Phylogenetic Relationship

We conducted phylogenetic analyses of all sampled individuals based on the nuclear genome SNPs. Consistenting with our earlier research (Wang et al., 2018), all samples of salt cress were clustered into four distinct lineages, Y1 to Y4 (Supplementary Figure 2; Supplementary Tables 1, 2). Y1 comprised all individuals from northern China (Y1), Y2 contained sampled individuals from western China (Xinjiang) while Y3 contained those from Altai. Y4 comprised all individuals from NE Russia and Canada.

### Local Selection in North America-Russia and Northern China

**Figure 3:**
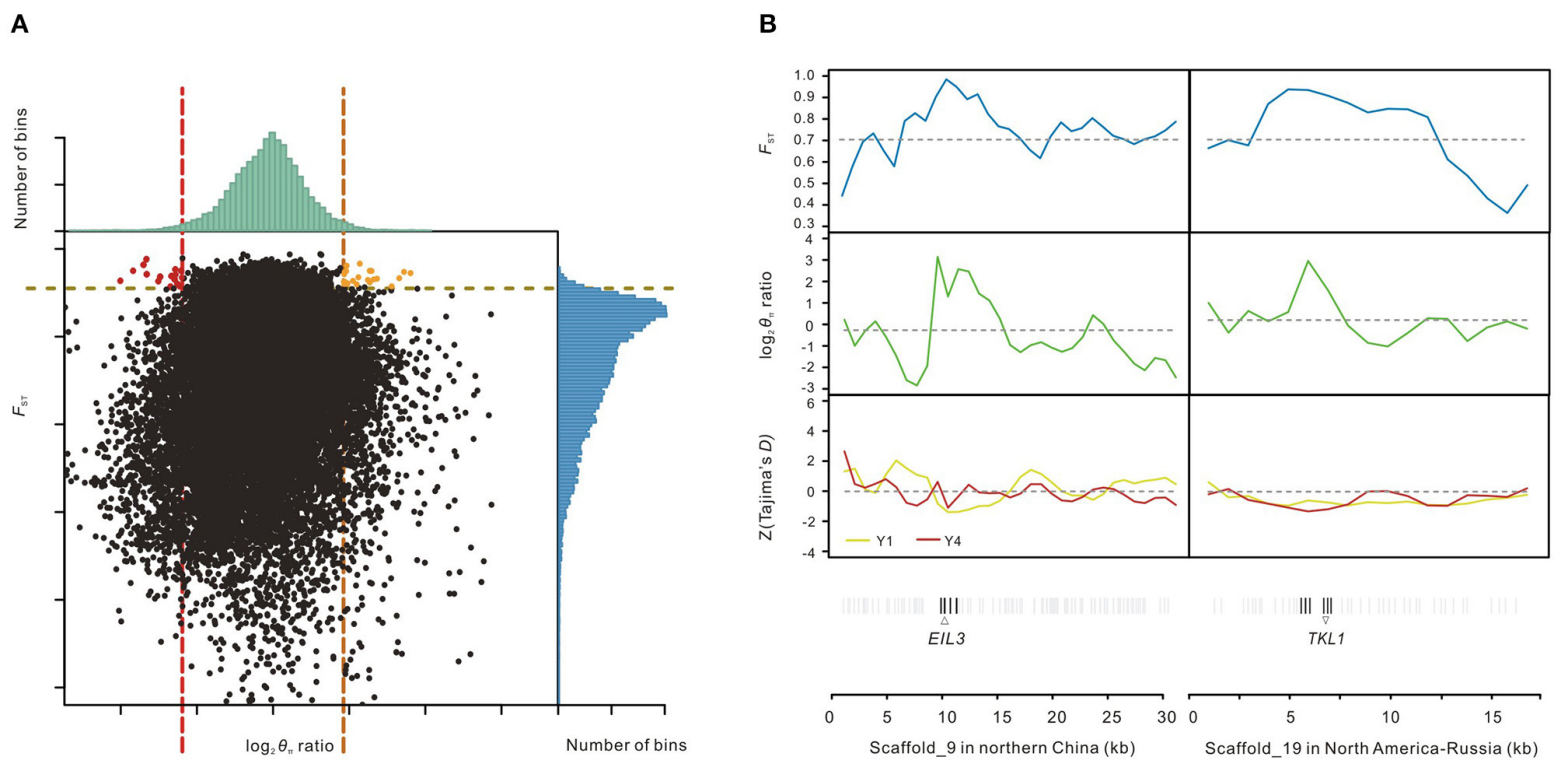
Identification of genomic regions with strong selective sweep signals in northern China and North America-NE Russia. **(A)** Distribution of log_2_ θ_π_ ratios Y1 (θ_π_, Y4/θ_π_, Y1), Y4 (θ_π_, Y1/θ_π_, Y4) and *F*_ST_ of 20 kb windows with 10 kb steps. Orange and red dots represent windows containing the selected regions of the northern China populations (Y1) and the North America-NE Russia populations (Y4), respectively (corresponding to 5% right tails of θ_π_ ratios distribution and 5% right tail of the *F*_ST_ distribution). **(B)** Two scaffolds with strong selection sweep signals in northern China and North America-NE Russia salt cress. *F*_ST_, log_2_ θ_π_ ratios and Tajima's *D* values are plotted using a 20 kb sliding window with 10 kb steps. Horizontal dashed lines represent mean whole-genome of corresponding values. Genes are shown at the bottom (black lines represent the putatively selected genes); Pale gray lines represent the other genes.

To detect accurately the genomic footprints left by local selections between two main salt cress ecotypes defined by phylogenetic analyses (Wang et al., 2018), we applied genetic diversity tests to data from the two groups of populations, Y1 (northern China) and Y4 (North America-NE Russia). Putatively selected genes (PSGs) were identified by screening selected windows simultaneously with significantly high log_2_ [Y1 ratio (θ_π_, Y4/θ_π_, Y1) and Y4 ratio (θ_π_, Y1/θ_π_, Y4)] (5% right tail, where log_2_ (Y1 ratio) is 1.84 and log_2_ (Y4 ratio) is 2.4043) and significantly high *F*_ST_ values (5% right tail, *F*_ST_ threshold: 0.9098) between them (Li et al., 2014; Qiu et al., 2015; Ma et al., 2018), which also exhibited significant differences (*p*-value <10^−16^, Mann-Whitney *U*-test) (Figure 3A). We identified 23 regions under selection for the population Y1 (northern China) with a total size of around 0.43 Mb (0.17% of the genome) and 17 regions for the population Y4 (North America-Russia) with a total size of around 0.33 Mb (0.13% of the genome). A total of 63 genes including 60 protein-coding genes and three hypothetical ones were annotated in the regions under selection of Y1 from northern China, while 42 protein-coding genes were annotated for those of Y4 from North America-Russia (Figure 3A; Supplementary Tables 4, 5). Functions of the genes from northern China are involved in DNA repair, plastid fission, meiosis, regulation of defense response to fungus, and regulation of sulfur metabolic process (Supplementary Table 4), while gene ontology (GO) enrichment analysis revealed that genes from North America-Russia are functionally related to response to various carbohydrates, transport, peptide biosynthetic process, and long-chain fatty acid biosynthesis process (Supplementary Table 5). Example of genes with strong selection sweep signals in northern China and North America-Russia are shown in Figure 3B.

## Discussion

### Species-Wide Selective Sweep

Salt cress can tolerate salt concentrations up to 500 mmol/L NaCl (Zhu, 2001), allowing it to survive on soils with high salt content. Such habitats would be expected to exert a high selection pressure and reduce genetic diversity in genomic regions involved in salt resistance. Since salt cress is a selfing species in which a high level of linkage disequilibrum is expected, these selective effects could also extend well-beyond the genomic regions under selection. Indeed, LD in salt cress is higher than those of other species examined (Wang et al., 2018) and its genetic diversity is extremely low and similar to those of domesticated rice and soybean (Wang et al., 2018). Therefore, regions showing lower diversity appeared more likely to be under selection pressure (lower Tajima's D, Pearson correlation efficient = 0.516, *p* <2.2e-16), further suggesting that such regions resulted more from selection pressure than from genetic drift or other neutral demographic processes.

Abiotic stress tolerances of plants are likely to involve in several different physiological and developmental pathways, such as osmotic homeostasis, stress damage control and repair, and growth regulation (Zhu, 2001, 2002, 2003; Mahajan and Tuteja, 2005). Some genes among the 227 annotated genes for which selective sweeps were detected operate in pathways important in salt tolerance (Table 1; Supplementary Table 3). For example, *ACO2* is a key member of the ethylene synthesis pathway, while *ERF13* is an important ethylene-responsive transcription factor (Schellingen et al., 2014; Sogabe et al., 2014). Ethylene-mediated signaling pathways have been shown to be critically involved in enhanced salt tolerance in plants (Ryu and Cho, 2015). In addition, *MDAR2* and *NDPK1*, are related to the removal of toxic H_2_O_2_ (Fukamatsu et al., 2003; Lisenbee et al., 2005), while *NUDT7* plays a vital role in regulating redox homeostasis during salt stress/defense signaling and programmed cell death in plant disease resistance (Muthuramalingam et al., 2015). Finally, several annotated genes are involved in growth regulation: for instance, *SCR*, a GRAS family transcription factor, regulates stem cell fate of the immediately surrounding cells (Moreno-Risueno et al., 2015), while *PYK10* encodes b-glucosidase in the sub-cellular compartments after wounding (Hara-Nishimura and Matsushima, 2003).

### Local Selections in North America-Russia and Northern China

Local selections by the different habitats plays a fundamental role in the production and maintenance of genetic diversity (Savolainen et al., 2013). In the middle Pleistocene, the climate tended to become drier and cooler while desertification and salinization began to develop and expand in central Asia (Wang et al., 2015). These changes might have triggered origin of *E. salsugineum* and its divergence into northern China and NE Russia-North America via two long-distance dispersal ways (Wang et al., 2018), where may be important in local adaptation selected by their different habitats. Functional enrichment analysis of GO terms revealed a remarkable amount of divergence between salt cress populations in northern China and North America-Russia, suggesting genome-wide selection by these local habitats (Figure 3; Supplementary Tables 4, 5).

The acclimated freezing tolerance of salt cress was positively correlated with the average minimum habitat temperature (Yang et al., 2013), similar to those of *Arabidopsis* (Hannah et al., 2006; Zhen and Ungerer, 2008). The average minimum habitat temperatures during the coldest month of the growing season at collection sites in North America and Northeast Russia are lower 10 degrees below zero, while that at collection sites in northern China are above zero (Yang et al., 2013). The Yukon cress ecotype from North America-Russia can even tolerate temperatures as low as −19°C (Griffith et al., 2007). Low temperatures induce a number of alterations in cellular components, including the amount of unsaturated fatty acids (Mahajan and Tuteja, 2005) and changes in protein and carbohydrate composition (Lynch and Thompson, 1982). The accumulation of sucrose and other simple sugars that occurs with cold acclimation also contributes to the stabilization of membranes as these molecules protect membranes against freeze-damage (Mahajan and Tuteja, 2005). This was confirmed by the enrichment and classification of GO terms of genes exhibiting selection signals. Most of these annotated genes are involved in carbohydrate stimulus, peptide biosynthetic and unsaturated fatty acids metabolic process (Supplementary Table 5). For example, *ADS2* is critical in the synthesis of unsaturated fatty acids that are an essential component for cold adaptation (Chen and Thelen, 2013) while the gene *STH1* (Figure 2B), as the homolog of the Salt Tolerance protein (STO) in *Arabidopsis*, may similarly regulate photomorphogenesis in light signaling in response to low temperatures (Salazar, 2010). In addition, a homolog of two tandemly duplicated genes *TKL1* (Figure 2B) in cucumber (*CsTK*) increases both photosynthetic rate and carboxylation efficiency under low temperature and light intensity (Bi et al., 2013). Both *STH* and *TKL1* genes exhibited the higher expressions in Yukon cress ecotype from North America-Russia than in Shandong cress ecotype from northern China when they were grown in the low-temperature common garden or in cabinet (Lee et al., 2012). These findings suggest that salt cress variants found at high latitude in North America and NE Russia could be associated with adaptation to low-temperature habitats.

In northeast China, salt cress usually grows close to vast flood plains with high salinity, extremely wet air and high chemical pollution compared with other populations (Wang et al., 2015). Contaminated soil is a great threat for plants, which can even cause damage to DNA. As a predominantly selfing plant, rapid habitat range expansion could bring about a large amount of harmful mutations (Wang et al., 2018). It is likely that many of these selected DNA-repair associated genes could efficiently clear up the negative effects of exposure of harmful varients. Two of the over-represented gene ontology categories in northern Chinese populations were “telomere maintenance in response to DNA damage” and “DNA recombination” (Supplementary Table 4). Some of these genes, for example, *IPT7*, are involved in cytokinin (CK) biosynthetic process that promotes cell differentiation and regulates root length (Dello Ioio et al., 2012), while *RAD54* is an important eukaryotic-specific recombination factor that plays a critical role in repairing damaged DNA due to radiation or heavy metal contamination (Sung, 1994; Raoul Tan et al., 2003). Moreover, only did it reach northern China, salt cress expanded and reached widespread distributions (Wang et al., 2018) might due to temperate climate conditions along the inland of the Yellow River and stronger resistance to pathogens (Yeo, 2014). Some other selected genes are related to defense against pathogens including “regulation of defense response to fungus” and “regulation of sulfur metabolic process.” Two genes, *SPLAYED* and *EIL3* (Figure 3B), are known to play critical roles in enhancing defense against pathogens in biotic stress signaling networks (Van der Ent et al., 2008; Walley et al., 2008). In addition, the gene *WES1* plays a key role in plant sulfur metabolism such as auxin and phytoalexin camalexin biosynthesis in pathogen stress response (Wang et al., 2012). Undoubtedly, genome-wide adaptive divergence further support that salt cress of northern China could display good resistance to biotic stress in their nature habitat (Pedras and Zheng, 2010; Yeo, 2014).

Due to its natural adaptations to various harsh climates and soil conditions, *Eutrema salsugineum* has long been used as an important model for deciphering mechanisms of salt and other abiotic stress in plants (Gong et al., 2005; Griffith et al., 2007; Lamdan et al., 2012). During adaptive evolution of the salt cress, genetic variation and natural selection are non-randomly fostering stress-related genes, gene interaction network in the whole genome, as well as prompting local adaptation, differentiation and diverse biological stresses between different accessions.

## Data Availability Statement

Publicly available datasets were analyzed in this study. This data can be found here: All Illumina sequence data would be deposited in the National Center for Biotechnology Information short-read archive (project SRP135200).

## Author Contributions

XW and GZ participated in the design of this study. XW and HR performed the statistical analysis. XC collected important background information. JM provided assistance for data analysis. XW wrote the manuscript. GL and GZ revised the manuscript. All authors contributed to the article and approved the submitted version.

## Conflict of Interest

The authors declare that the research was conducted in the absence of any commercial or financial relationships that could be construed as a potential conflict of interest.

## Publisher's Note

All claims expressed in this article are solely those of the authors and do not necessarily represent those of their affiliated organizations, or those of the publisher, the editors and the reviewers. Any product that may be evaluated in this article, or claim that may be made by its manufacturer, is not guaranteed or endorsed by the publisher.
