# Supplementary material for: Genomic Variation Landscape of the Model Salt Cress Eutrema salsugineum
Source: Front Plant Sci. 2021 Aug 17;12:700161. doi: 10.3389/fpls.2021.700161 (PMC8416042; doi:10.3389/fpls.2021.700161)
Supplement: Supplementary file 2 [file Data_Sheet_2.doc]

Supplementary Information for

**Genomic variation landscape of the model salt cress *Eutrema*** ***salsugineum***

**Supplementary Figures**


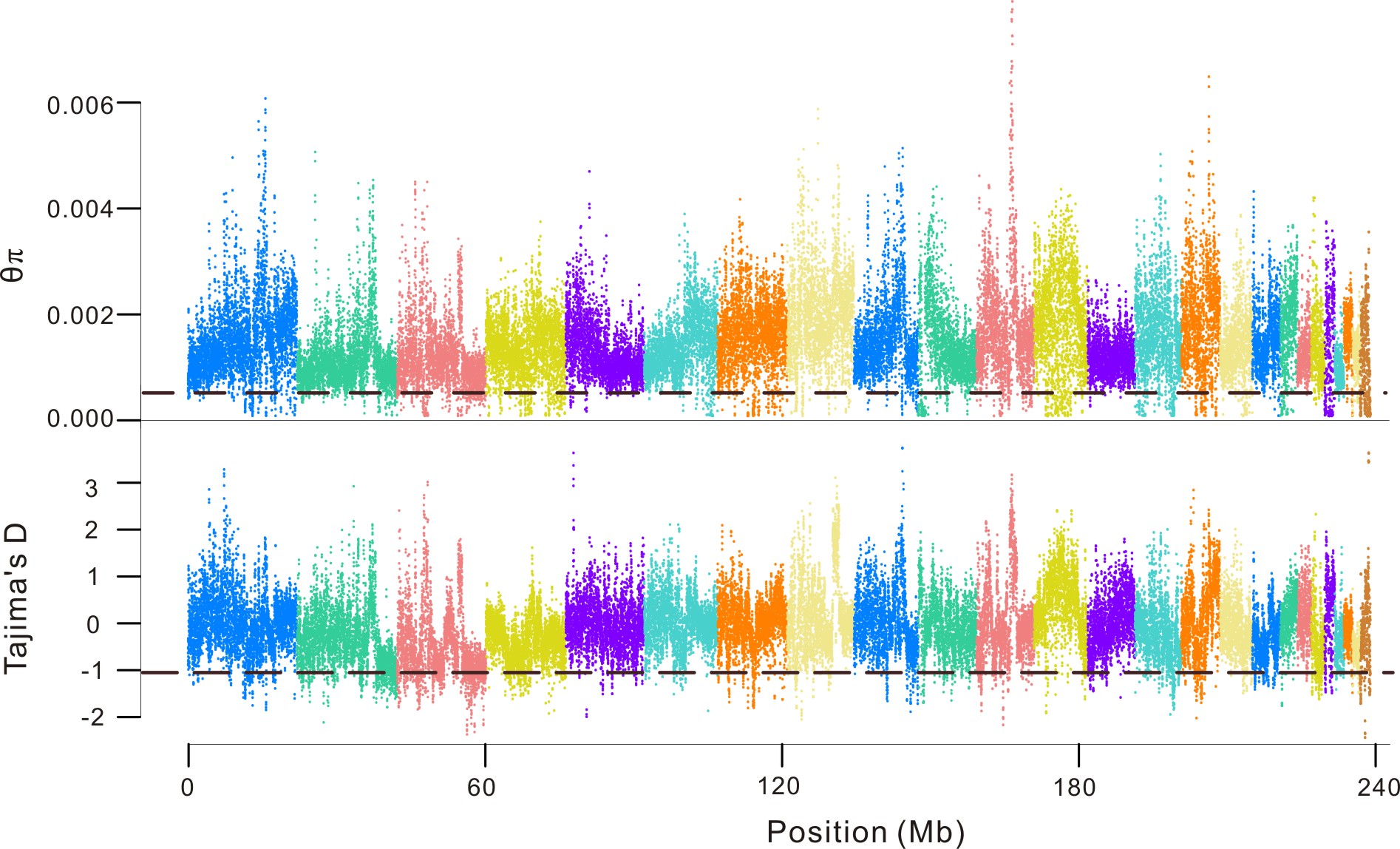


**Fig S1.** Genome-wild distribution of θπ and Tajima’s D in the entire data, which are calculated in 20 kb windows sliding in 10 kb steps. The horizontal dashed line indicates the threshold defining the selective sweeps (θπ ≤0.00053 and Tajima’s D ≤-1.0197).


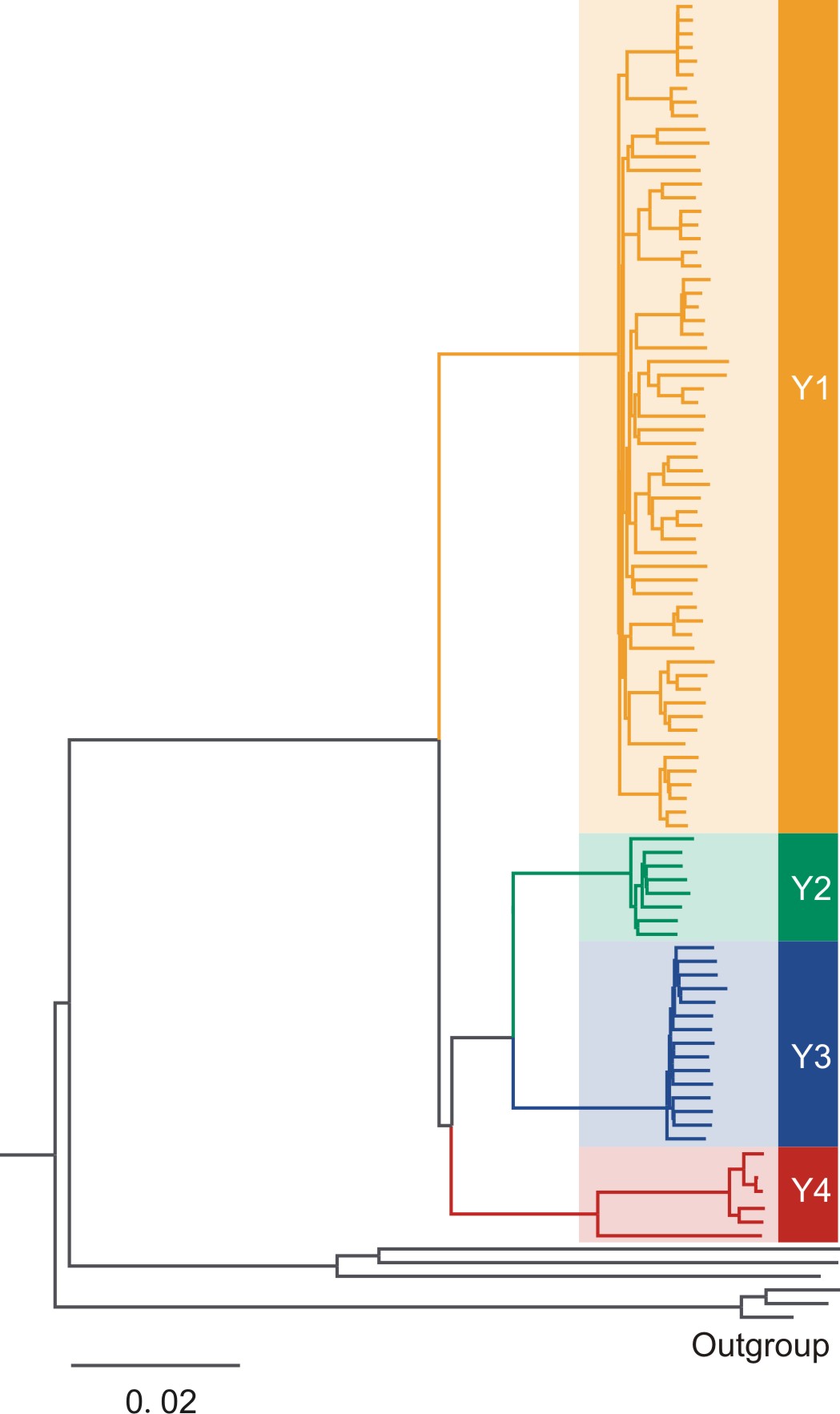


**Fig S2.** Phylogenetic analyses of the salt cress. A rooted phylogenetic tree conducted using whole-genome SNPs data, with *E. halophilum* and *E. botschantzevii* as outgroups. The scale bar represents the *p* distance. Y1: populations from northern China; Y2: the population from western China (Xinjiang); Y3: the population from Altai salt; and Y4: populations from North America and Russia.
